# Soluble Isoforms of Vascular Endothelial Growth Factor Are Predictors of Response to Sunitinib in Metastatic Renal Cell Carcinomas

**DOI:** 10.1371/journal.pone.0010715

**Published:** 2010-05-19

**Authors:** Bernard Paule, Laurence Bastien, Emmanuelle Deslandes, Olivier Cussenot, Marie-Pierre Podgorniak, Yves Allory, Benyoussef Naïmi, Raphael Porcher, Alexandre de La Taille, Suzanne Menashi, Fabien Calvo, Samia Mourah

**Affiliations:** 1 Inserm, UMRS 940, Paris, France; 2 AP-HP, Hôpital Henri Mondor, Department of Urology, Inserm-U841Eq07, Créteil, France; 3 Inserm, U717, Department of Biostatistics and Medical Data Processing, Université Paris 7, Hôpital Saint-Louis AP-HP, Paris, France; 4 AP-HP, Hôpital Tenon, Department of Urology and the CeRePP National Group, Paris, France; 5 AP-HP, Hôpital Saint-Louis, Laboratory of Pharmacology, Paris, France; 6 CNRS-UMR 7149, Université Paris Est, Créteil, France; 7 Université Paris 7- Denis Diderot, Paris, France; Universität Heidelberg, Germany

## Abstract

**Background:**

Angiogenesis is the target of several agents in the treatment of malignancies, including renal cell carcinoma (RCC). There is a real need for surrogate biomarkers that can predict selection of patients who may benefit from antiangiogenic therapies, prediction of disease outcome and which may improve the knowledge regarding mechanism of action of these treatments. Tyrosine kinase inhibitors (TKI) have proven efficacy in metastatic RCC (mRCC). However, the molecular mechanisms underlying the clinical response to these drugs remain unclear.

**Methodology/Principal Findings:**

The present study aimed to identify molecular biomarkers associated with the response to sunitinib, a Tyrosine kinase inhibitor. To evaluate this relationship, primary tumors from 23 metastatic RCC patients treated by sunitinib were analyzed for a panel of 16 biomarkers involved in tumor pathways targeted by sunitinib, using real-time quantitative reverse-transcriptase PCR. Nine of the 23 patients (39%) responded to sunitinib. Among transcripts analyzed, only the levels of vascular endothelial growth factor (VEGF) soluble isoforms (VEGF_121_ and VEGF_165_) were associated with the response to sunitinib (*P* = 0.04 for both). Furthermore, the ratio of VEGF soluble isoforms (VEGF_121_/VEGF_165_) was significantly associated with prognosis (*P* = 0.02).

**Conclusions:**

This preliminary study provides a promising tool that might help in the management of metastatic RCC, and could be extended to other tumors treated by TKI.

## Introduction

Renal cell carcinoma (RCC) represents 5% of malignancies with 38,000 new cases diagnosed in 2006 in the United States [1]. During last decades, this incidence has constantly increased. At the time of diagnosis, about 30% of RCC are metastatic. A genomic deletion, involving the von Hippel Lindau (VHL) gene is common in clear-cell RCCs, which represents 75% of RCCs. Both alleles of the VHL suppressor gene are inactivated either by deletion, mutation, or promoter hypermethylation [2].The alteration of VHL leads to an anarchic stimulation of hypoxic response due to a dysregulation of α subunits of hypoxia inducible factor (HIF). The stimulation of HIF results in a dysregulation of HIF target genes, mainly those encoding for the vascular endothelial growth factor (VEGF), its receptors (VEGFR), the platelet-derived growth factor (PDGF) and the urokinase-type plasminogen activator (uPA) with consequences on angiogenesis and invasion [3]. Angiogenesis plays an important role in the invasion and dissemination of RCC, and is mediated by numerous factors. Among pro-angiogenic factors, VEGF is the mainstay of this process [3], [4]. VEGF has five main isoforms produced by alternative splicing of a gene located on 6p21.3: VEGF_121_, VEGF_165_, VEGF_189_, VEGF_145,_ and VEGF_206_, which differ in their bioavailability [5].

Among new concepts developed to improve the management of metastatic RCC (mRCC), molecules targeting the VEGF have been developed, especially tyrosine kinase inhibitors (TKI). In first-line therapy, sunitinib significantly improved progression-free survival by reducing the risk of relapse by 58% compared with interferon-α [6]. To date, no predictive biological factors of response have been identified allowing a better selection of RCC patients for sunitinib therapy. The present study aimed to identify biomarkers associated with sunitinib response. To evaluate this relationship, primary tumors from 23 clear-cell metastatic RCC patients treated by sunitinib were analyzed retrospectively for the expression of a biomarker panel involved in tumor pathways targeted by sunitinib.

## Results and Discussion

Primary tumors from 23 metastatic RCC patients treated by sunitinib were analyzed for the gene expression of a panel of 16 biomarkers involved in tumor pathways targeted by sunitinib, using real-time quantitative reverse-transcriptase PCR (qRT-PCR). A correlation between their expression and the response to sunitinib was then evaluated.

According to RECIST criteria [6], [7], overall objective response to sunitinib was achieved in 18 patients. Partial response (PR) was observed in 9 patients and response of stable disease (SD) ≥3 months were observed in 9 patients. No complete response was observed. The median duration of follow-up was 26.6 months.

**Figure 1:**
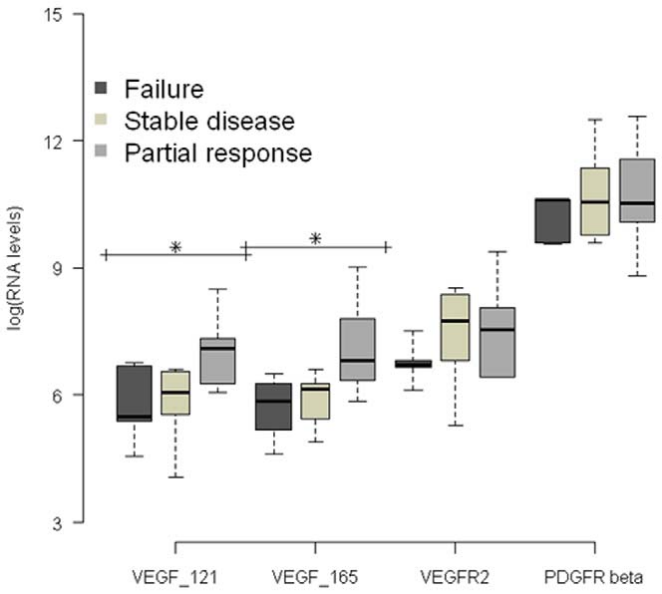
Boxplot of four RNA expression levels (in log_10_ scale) according to treatment response. The expression levels of interesting transcripts were normalized to the housekeeping PPIA (peptidylprolyl isomerase A) and TBP (TATA-box binding protein) gene transcripts. Since there was no difference between control genes, results were presented as copies of target gene per copy of PPIA. The median values and their corresponding logarithmic values in brackets were respectively: for VEGF_121_ PR 1222 (7.1), SD 425 (6.0) and Failure 241 (5.4); and for VEGF_165_ PR 905 (6.8), SD 460 (6.1) and Failure 352 (5.8). (*) for p<0.05.

Soluble VEGF isoforms, VEGF121 and VEGF165, were significantly associated with the response to sunitinib at three months (Figure 1). Indeed, the tumor transcript levels were significantly higher in responding (RP and SD) patients compared with patients who had a failure to treatment. The median values were respectively: for VEGF_121_ PR: 1222; SD: 425; Failure: 241 (p = 0.04), and for VEGF_165_ PR: 905; SD: 460; Failure: 352 (p = 0.04). For tumors overexpressing VEGF_121_ and VEGF_165_, the probability of response was 81% and 90%, respectively.

**Figure 2:**
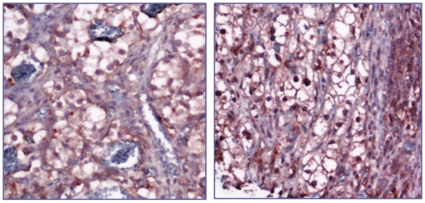
Immunohistochemical staining of total VEGF protein in sections of human RCC tissues. Representative RCC tumor center (left) and margins (right) with lower and strong expression of VEGF respectively.

The immunohistochemical study of total VEGF protein (using a human-anti-VEGF antibody R&D, France) showed a significant correlation between tumor response and difference in VEGF expression between tumor center and margins (p = 0.015). Indeed, the higher was this difference, the better was the response (Figure 2). No significant correlation was found between mRNA levels of VEGFR-1, VEGFR-2 and PDGF-Receptors (targeted by sunitinib), and response to sunitinib.

**Figure 3:**
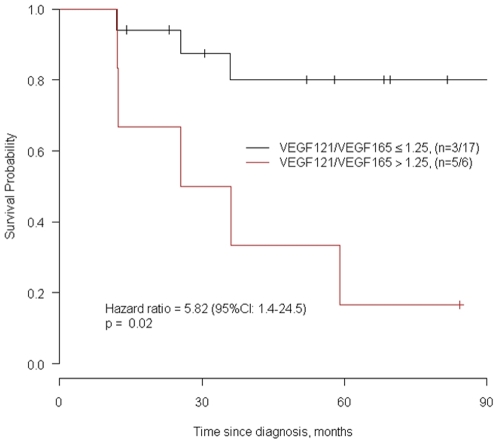
Kaplan-Meier survival curve according to the ratio VEGF_121_/VEGF_165._

The response to sunitinib was independent from tumor size and noteworthy, from the prognostic group and was not significantly associated with Fuhrman grade. Furthermore, the overall survival of patients with a VEGF_121_/VEGF_165_ ratio lower than 1.25 (ratio cut-off value determined from the third quartile) was significantly higher than those of patients with a ratio higher than 1.25 (p = 0.02; median survival time, 25.2 months *versus* not reached). Indeed, as evaluated by a Cox proportional cause specific hazards model, the estimated hazard ratio (HR) for the risk of death since diagnosis in the group with high values of VEGF_121_/VEGF_165_ ratio was 5.8 (95 percent confidence interval: 1.4 to 24.5; p = 0.02) (Figure 3).

Transcript levels for VEGF_121_ and VEGF_165_ were significantly higher for the subset of patients with Fuhrman grade 1 or TNM stage 1 tumors (p = 0.05; p = 0.04 and p = 0.003; p = 0.002 respectively). When considering treatment effect, sunitinib partial response was found to decrease the incidence rate by 50% though this was not significant. (HR: 0.50, 95% CI: 0.10 to 2.50, p = 0.40) and by 73% for stable disease (HR: 0.27, 95% CI: 0.04 to 1.63, p = 0.15). The ratio remained non significant when considering pooled data (failure versus PR and SD). The estimated hazard ratio in the partial response and stable disease group is 0.37 (95% CI: 0.09 to 1.59; p = 0.18).

To date, no predictive factor or biomarker of the response to sunitinib has been identified in mRCC. Moreover, there are no clinical factors that can discriminate and/or predict a preferential efficacy of TKIs compared with mTOR inhibitor or bevacizumab. Some trends were observed such as slight differences according to age [8], and histological subtypes [9]. The occurrence of arterial hypertension has been evoked as a predictive factor of response to sunitinib [10]. In terms of biomarkers, patients with either high or low baseline plasma VEGF benefit from treatment in terms of progression-free survival in a large cohort of mRCC treated with sorafenib [11]. In another study, Fold increase in plasma VEGF after two cycles of sunitinib treatment in clear cell-RCC was shown to be significantly lower in patients that obtained clinical benefit as compared to patients that progressed [12]. This biomarker measurement allows therefore the stratification of patients in respect to response only after two cycles of treatment. In a series of five mRCC patients, the free-plasma VEGF has also been found to be a possible pharmacodynamic marker for bevacizumab antiangiogenic activity [13]. It has been also demonstrated that sunitinib inhibits signaling pathways involved in bevacizumab resistance in mRCC patients, and that baseline levels of soluble VEGFR-3 and VEGF-C may have a potential utility as biomarkers of clinical efficacy in this setting [14]. These findings support the potential significance of the VEGF/VEGFR-2 pathway predominant in mRCC. In spite of the inhibition of VEGFR-2 by sunitinib, the magnitude of its role *in vivo* is not fully clarified. Clinically, patients who have high levels of VEGF soluble isoforms achieve a better response to sunitinib. Thereby, the VEGF/VEGFR pathway could be the preferential target of sunitinib. Our results favored the hypothesis that response to therapy is associated with the inhibition of VEGF pathway, depending on the inhibition of VEGF signal. This is likely due to the inhibition of both angiogenesis and cell proliferation driven by the presence of a VEGF/VEGFR-2 autocrine loop in tumor cells. Indeed, RCC tumor cells, producing high levels of VEGF_121_ and VEGF_165_, display a higher ability to grow and to invade the extracellular matrix [15], [16]. Based on these observations, further researche confirming clinical significance and underlying biologic mechanisms are warranted.

Regarding the prognostic value, serum VEGF protein levels are prognostic for progression-free and overall survivals in RCC [11]. Patients with higher tumor VEGF_121_ mRNA levels have a significantly shorter survival compared with those having lower levels suggesting that angiogenic activity might be up-regulated in tumors with a high ability to invade [17], [18]. Thereby, patients with high soluble VEGF levels might have a more aggressive disease, and the improved outcome observed in our series might be a reflection of disease biology.

It is hard to speculate at this point on why the association of survival is better with the ratio of VEGF_121_/VEGF_165_ than with VEGF_121_ alone. However, we have previously shown that VEGF_121_ is the most expressed isoform in RCC [17]. Tomisawa et al have also shown that VEGF_121_ is more expressed than VEGF_165_ and VEGF_189_ and while all the analyzed RCC primary tumors expressed VEGF_121_, only 70% showed expression of VEGF_165_. In addition, these authors report that neither VEGF_165_ nor VEGF_189_ were expressed alone in RCCs [16]. It is worth noting that this ratio avoids the use of housekeeping genes and would thus provide a more easy to use clinical test.

Therefore, if these new drugs provide considerable promise for patients, there is a crucial need for a better selection of patients. Indeed, tumors with close characteristics can present opposite behavior with either important and long regressions, or very short-term progressions. TKI are multikinase inhibitors, which impact a wide cascade of signaling pathways. Thereby, the optimization of their efficiency is based on a correlation between response to treatment and individual tumor signatures.

In conclusion, this preliminary study constitutes a first step in the identification of surrogate biomarkers of sunitinib antiangiogenic activity in mRCC and requires confirmation in a larger independent series of patients. Indeed, tumor soluble VEGF mRNA represents a potentially promising tool that might help the clinician to identify patients who are likely to benefit from sunitinib and avoid a costly and potentially toxic administration of this treatment in non-responding patients.

## Materials and Methods

### Patients

We analyzed retrospectively data from 23 consecutive patients with clear-cell mRCC, and treated in a single institution (Hôpital Henri Mondor, Créteil, France) with sunitinib (delivered orally at a dose of 50 mg/day for 4 weeks, every 6 weeks) after failure of a first-line therapy with interferon-α. The study was performed in accordance with the precepts established by the Helsinki Declaration and approved by Hôpital Henri Mondor Ethic Committee; patients were enrolled after giving written consent. All data were analyzed anonymously. Patients' characteristics are summarized in Table 1.

**Table 1:** Patient Characteristics.

Characteristics	Number of patients	Percentage
Gender		
Male	16	69.6
Female	7	30.4
TNM stage at diagnosis		
1 (T1a/T1b)	4	17.4
2 (T2)	5	21.7
3 (T3a/T3b)	14	60.9
Fuhrman grade		
1	3	13.1
2	5	21.7
3	7	30.4
4	8	34.8
ECOG Performance Status		
0	19	82.6
1	4	17.4
Sites of metastases*		
Lung	15	48.4
Bone	6	19.3
Lymph nodes	5	16.1
Other (liver, kidney)	5	16.1
MSKCC risk factor		
Favorable	10	43.5
Intermediate	11	47.8
Poor	2	8.7
Treatment response at three months		
Failure	5	21.7
Partial response (PR)	9	39.1
Stable disease (SD)	9	39.1

MSKCC: Memorial Sloan-Kettering Cancer Center; ECOG: Eastern Cooperative Oncology Group.

*Several sites per patient.

Tumor response was assessed according to the Response Evaluation Criteria in Solid Tumor (RECIST) after three months of sunitinib treatment [6], [7].

### Biomarkers Evaluation

A panel of 16 biomarkers involved in angiogenesis and invasion pathways was assessed. The transcript panel included VEGF (isoforms 121, 165, and 189), and their receptors (VEGFR-1 and R-2); PDGF-A and -B and their receptors (PDGF-Rα and -Rβ); fibroblast growth factor (FGF)-2; HIF-1α; chemokine receptor 4 (CXCR4); uPA, its receptor (uPA-R) and inhibitor plasminogen activator inhibitor-1 (PAI-1); and lymphatic vessel endothelial receptor-1 (LYVE-1), an extracellular-matrix transmembrane receptor.

Samples from primary tumors of clear-cell RCC not containing necrosis were selected and the different biomarker analyses were performed on adjacent sections.

Total RNA was extracted from frozen tumors using TRIzol (Invitrogen). cDNA was synthesized using High-Capacity cDNA Kit (Applied-Biosystems). Transcript levels were measured in each tumor by quantitative RT-PCR using Perfect-Master Mix-Probe (AnyGenes, France) on LightCycler (Roche, France). The expression levels of interesting transcripts were normalized to the housekeeping PPIA (peptidylprolyl isomerase A) and TBP (TATA-box binding protein) gene transcripts. Since there was no difference between control genes, results were presented as copies of target gene per copy of PPIA. Gene set assays were designed using Primer-Express Software (Applied-Biosystems) and primers and probes sequences were available upon request. Gene expression levels were determined using standard calibration curves prepared from gene-specific PCR products. All PCRs were done in duplicate.

Immunohistochemical analyses were carried out using antibodies directed against VEGF (Abcam), VEGF-R1, VEGF-R2 (R&D Systems), and PDGF-Rβ (Cell Signaling). Tissue sections were incubated overnight with the specified primary antibody, and then incubated with the appropriate biotinylated secondary antibodies. Peroxidase reactivity was visualized using 3-amino-9-ethylcarbazole (AEC, DAKO).

### Statistical Analysis

Variables analyzed were according to the Memorial Sloan-Kettering Cancer Center (MSKCC) risk model, Fuhrman grade, and treatment response at three months. Gene expression levels were presented in log10 scale. Mann-Whitney or Kruskal-Wallis tests were used. Survival curves were estimated by the Kaplan-Meier method, and compared using a log-rank test. The characteristics associated with the risk of dying were tested using a Cox proportional cause-specific hazards model. The association between biomarkers and the risk of dying was reported as the hazard ratio (HR) together with its 95% confidence interval (95%CI). All tests and p-value were two-sided, and differences were considered as significant for p<0.05. Statistical analysis was performed using the Open Source R software (R 2.4.0).

## References

[1] Jemal A, Siegel R, Ward E, Murray T, Xu J (2006). Cancer statistics, 2006.. CA Cancer J Clin.

[2] Rini BI, Small EJ (2005). Biology and clinical development of vascular endothelial growth factor-targeted therapy in renal cell carcinoma.. J Clin Oncol.

[3] Amato RJ (2005). Renal cell carcinoma: review of novel single-agent therapeutics and combination regimens.. Ann Oncol.

[4] Carmeliet P (2003). Angiogenesis in health and disease.. Nat Med.

[5] Ferrara N, Gerber HP, LeCouter J (2003). The biology of VEGF and its receptors.. Nat Med.

[6] Motzer RJ, Hutson TE, Tomczak P, Michaelson MD, Bukowski RM (2007). Sunitinib versus interferon alfa in metastatic renal-cell carcinoma.. N Engl J Med.

[7] Therasse P, Arbuck SG, Eisenhauer EA, Wanders J, Kaplan RS (2000). New guidelines to evaluate the response to treatment in solid tumors. European Organization for Research and Treatment of Cancer, National Cancer Institute of the United States, National Cancer Institute of Canada.. J Natl Cancer Inst.

[8] Eisen T, Oudard S, Szczylik C, Gravis G, Heinzer H (2008). Sorafenib for older patients with renal cell carcinoma: subset analysis from a randomized trial.. J Natl Cancer Inst.

[9] Choueiri TK, Plantade A, Elson P, Negrier S, Ravaud A (2008). Efficacy of sunitinib and sorafenib in metastatic papillary and chromophobe renal cell carcinoma.. J Clin Oncol.

[10] Rixe O, Billemont B, Izzedine H (2007). Hypertension as a predictive factor of Sunitinib activity.. Ann Oncol.

[11] Escudier B, Eisen T, Stadler WM, Szczylik C, Oudard S (2009). Sorafenib for treatment of renal cell carcinoma: Final efficacy and safety results of the phase III treatment approaches in renal cancer global evaluation trial.. J Clin Oncol.

[12] Kontovinis LF, Papazisis KT, Touplikioti P, Andreadis C, Mouratidou D (2009). Sunitinib treatment for patients with clear-cell metastatic renal cell carcinoma: clinical outcomes and plasma angiogenesis markers.. BMC Cancer.

[13] Loupakis F, Falcone A, Masi G, Fioravanti A, Kerbel RS (2007). Vascular endothelial growth factor levels in immunodepleted plasma of cancer patients as a possible pharmacodynamic marker for bevacizumab activity.. J Clin Oncol.

[14] Rini BI, Michaelson MD, Rosenberg JE, Bukowski RM, Sosman JA (2008). Antitumor activity and biomarker analysis of sunitinib in patients with bevacizumab-refractory metastatic renal cell carcinoma.. J Clin Oncol.

[15] Takahashi A, Sasaki H, Kim SJ, Tobisu K, Kakizoe T (1994). Markedly increased amounts of messenger RNAs for vascular endothelial growth factor and placenta growth factor in renal cell carcinoma associated with angiogenesis.. Cancer Res.

[16] Tomisawa M, Tokunaga T, Oshika Y, Tsuchida T, Fukushima Y (1999). Expression pattern of vascular endothelial growth factor isoform is closely correlated with tumour stage and vascularisation in renal cell carcinoma.. Eur J Cancer.

[17] Rivet J, Mourah S, Murata H, Mounier N, Pisonero H (2008). VEGF and VEGFR-1 are coexpressed by epithelial and stromal cells of renal cell carcinoma.. Cancer.

[18] Tsuchiya N, Sato K, Akao T, Kakinuma H, Sasaki R (2001). Quantitative analysis of gene expressions of vascular endothelial growth factor-related factors and their receptors in renal cell carcinoma.. Tohoku J Exp Med.

